# Oral manifestations in a boy with X-linked reticulate pigmentary disorder

**DOI:** 10.1186/1746-160X-8-S1-P9

**Published:** 2012-05-25

**Authors:** M Callea, M Maglione, I Yavuz, L Deroma, CE Willoughby, G Tadini

**Affiliations:** 1 Department of Maxillo-Facial Surgery and Paediatric Dentistry, Institute for Maternal and Child Health, Trieste, Italy; 2 University of Trieste, Italy; 3 Dicle University, Diyarbakır, Turkey; 4 University Hospital Santa Maria della Misericordia, Udine, Italy; 5 Queen’s University Belfast, Northern Ireland, UK; 6 University of Milano, Italy

## Introduction

X-linked reticulate pigmentary disorder (XLPDR) is a rare, multi-systemic disease with only a limited number of families described in the literature. XLPDR has a genetic origin and the gene has been mapped to Xp22-p21. Dental features resemble those of hypohidrotic ectodermal dysplasia.

## Case report

A 3-year-old boy was seen at the Department of Maxillo-Facial Surgery and Paediatric Dentistry of the Children’s Hospital of Trieste. The multi-systemic features of XLPDR included a number of oro-dental manifestations such as misshapen teeth, scissor bite, swallowing difficulties, agenesis of the 4 permanent second premolars, taurodontism, early resorption of deciduous roots, and premature tooth eruption in general. Other important characteristics are crowding of permanent inferior tooth crowns, severe gingivitis, enamel hypoplasia and discoloration due to multiple antimicrobial treatments.

## Discussion

XLPDR is a rare form of ectodermal dysplasia with multi-systemic manifestations requiring intensive medical supervision. Clinically, the oro-dental phenotype resembles that of hypohidrotic ectodermal dysplasia, but there are several novel, previously unreported features.

